# Defining the optimal sequence for the systemic treatment of metastatic breast cancer

**DOI:** 10.1007/s12094-016-1520-2

**Published:** 2016-06-17

**Authors:** J. A. Mestres, A. B. iMolins, L. C. Martínez, J. I. C. López-Muñiz, E. C. Gil, A. de Juan Ferré, S. del Barco Berrón, Y. F. Pérez, J. G. Mata, A. G. Palomo, J. G. Gregori, P. G. Pardo, J. J. I. Mañas, A. L. Hernández, E. M. de Dueñas, N. M. Jáñez, S. M. Murillo, J. S. Bofill, P. Z. Auñón, P. Sanchez-Rovira

**Affiliations:** 1 Hospital del Mar, Barcelona, Spain; 2 Hospital de la Santa Creu i Sant Pau, Barcelona, Spain; 3 Complejo Hospitalario Universitario de A Coruña, A Coruña, Spain; 4 Hospital Virgen de la Salud, Toledo, Spain; 5 Hospital Universitario 12 de Octubre, Madrid, Spain; 6 Hospital Universitario Marqués de Valdecilla, Santander, Spain; 7 Institut Català d’Oncologia, Girona, Spain; 8 Hospital Universitario Central de Asturias, Oviedo, Spain; 9 Hospital Santa María Nai en Ourense, Orense, Spain; 10 Complejo Asistencial de León, Léon, Spain; 11 Fundación Instituto Valenciano de Oncología, Valencia, Spain; 12 Hospital Universitari Vall d’Hebron, Barcelona, Spain; 13 Complejo Hospitalario de Navarra, Pamplona, Spain; 14 Hospital Clínic Universitari de València, València, Spain; 15 Consorcio Hospitalario i Provincial de Castellón, Castellón, Spain; 16 Hospital Universitario Ramón y Cajal, Madrid, Spain; 17 Hospital Universitari Arnau de Vilanova, Lleida, Spain; 18 Hospital Universitario Nuestra Señora de Valme, Seville, Spain; 19 Hospital Universitario La Paz, Madrid, Spain; 20 Complejo Hospitalario de Jaén, Jaén, Spain

**Keywords:** Metastatic breast cancer, Hormone therapy, Chemotherapy, Targeted therapies, HER2 receptor, Triple-negative tumor

## Abstract

Metastatic breast cancer is a heterogeneous disease that presents in varying forms, and a growing number of therapeutic options makes it difficult to determine the best choice in each particular situation. When selecting a systemic treatment, it is important to consider the medication administered in the previous stages, such as acquired resistance, type of progression, time to relapse, tumor aggressiveness, age, comorbidities, pre- and post-menopausal status, and patient preferences. Moreover, tumor genomic signatures can identify different subtypes, which can be used to create patient profiles and design specific therapies. However, there is no consensus regarding the best treatment sequence for each subgroup of patients. During the SABCC Congress of 2014, specialized breast cancer oncologists from referral hospitals in Europe met to define patient profiles and to determine specific treatment sequences for each one. Conclusions were then debated in a final meeting in which a relative degree of consensus for each treatment sequence was established. Four patient profiles were defined according to established breast cancer phenotypes: pre-menopausal patients with luminal subtype, post-menopausal patients with luminal subtype, patients with triple-negative subtype, and patients with HER2-positive subtype. A treatment sequence was then defined, consisting of hormonal therapy with tamoxifen, aromatase inhibitors, fulvestrant, and mTOR inhibitors for pre- and post-menopausal patien ts; a chemotherapy sequence for the first, second, and further lines for luminal and triple-negative patients; and an optimal sequence for treatment with new antiHER2 therapies. Finally, a document detailing all treatment sequences, that had the agreement of all the oncologists, was drawn up as a guideline and advocacy tool for professionals treating patients with this disease.

## Introduction

The annual incidence of breast cancer in Spain is around 27,000 cases [1], and it causes more than 6200 deaths per year [1, 2]. Metastatic breast cancer (MBC), in particular, is a disease that varies widely, depending on the site of metastasis and its aggressiveness. It may present de novo (6–10 % of breast cancers) or it may appear as recurrent disease (20–50 % of patients) [3]. It occurs in many forms, each associated with a better or poorer disease prognosis [4]. While the aim of breast cancer treatment in other stages is curative, the objectives in the metastatic stage are mainly palliative [3]. The goal, then, is to increase survival and symptom control, while minimizing toxicity. However, treatment guidelines for MBC are not yet clearly defined.

Selecting a systemic treatment for MBC is a complex process, in which different factors must be considered. Some parameters are associated with the disease itself, including hormone receptor and HER2 receptor status, tumor proliferation index, previous disease-free survival (DFS), response to previous treatments, tumor molecular signatures [5], and tumor load. Other aspects to consider include the personal characteristics of the patient, such as age, menopausal status, personal preferences, comorbidities, adverse effects of previous treatments, psychological, and socioeconomic factors, etc.

Proper knowledge of the different therapeutic options is necessary to establish optimal and homogeneous treatment sequences. Multiple clinical guidelines for breast cancer are available, such as ESMO-ABC2, SEOM, GEICAM, and ASCO, etc. [6–9]. However, there is some ambivalence about best treatment options for each line. For example, a patient with a HER2-negative, hormone receptor-positive tumor could be treated in the first line with several hormone treatments and with different chemotherapy drugs, such as taxanes, anthracyclines, vinorelbine, or capecitabine in combination with bevacizumab [5, 7, 8]. This wide spectrum, ideal for individualized treatment, can be counterproductive in terms of the uncertainty it generates. Indeed, two patients with similar biologic and clinical characteristics can be treated in opposite ways.

While considering the obvious constraints of a consensual document, it may be interesting to set down a series of general treatment recommendations. This document is a reflection of the working criteria of several oncology specialists active in this field in Spain.

## Methodology

On the occasion of the San Antonio Breast Cancer Congress (SABCC) in 2014, an international group of breast cancer specialists held a parallel meeting, the aim of which was to define, on the basis of several clinical cases, the different profiles of MBC patients who may be candidates for similar treatment regimens during the natural history of their disease. Some of the Spanish oncologists who had attended the first meeting met later to review the different patient profiles, and some subgroups were established.

**Fig. 1:**
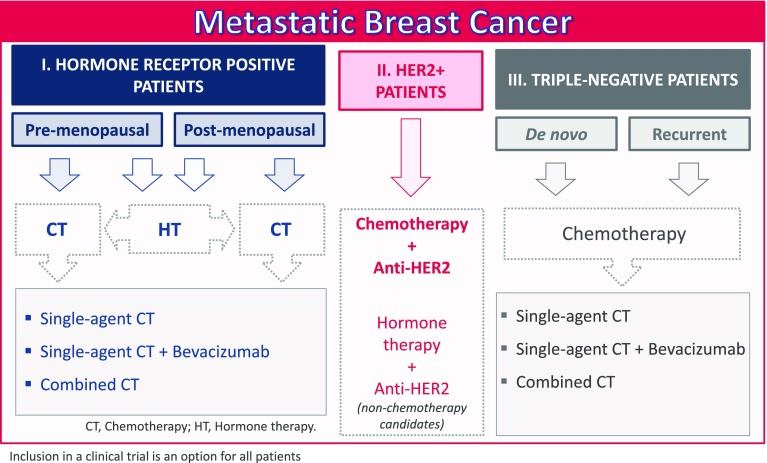
General treatment regimens for metastatic breast cancer

In this second meeting, the major patient profiles identified on the basis of a literature review were the following: breast cancer patients with positive hormone receptors (split into two subgroups, pre-menopausal and post-menopausal, with distinct hormone therapy approaches); patients with HER2-positive disease; and patients with triple-negative tumors (Fig. 1). The possibility of distinguishing between luminal A- and B-positive hormone receptors was discussed, but any differences were not determinant for the purpose of treatment sequencing, even if this information is useful for selecting the type of treatment (hormone therapy vs chemotherapy).

These proposals were discussed in a final meeting attended by a large group of Spanish breast cancer specialists, including the oncologists who had participated in the first two meetings. In this final meeting, the patient profiles were validated by all participant oncologists, who are the authors of this paper. Four working groups defined the most appropriate treatment sequences for each profile, which were subsequently validated by all participants, and the level of agreement was recorded.

Finally, the panel of breast cancer specialists drew up a document which was validated by all authors. Below is a summary of the agreed treatment sequences.

## Treatment option recommendations

There was no clear agreement on the preferential treatment sequence, although there is high agreement (95 %; 19/20) on favoring the sequential single-agent chemotherapy over combined chemotherapy in MBC. Combined chemotherapy is more toxic, and while the combination of several drugs may have more impact on the tumor, it remains unclear whether the lower doses and treatment time compared to monotherapy compensate for this increased toxicity [10].

The criteria for treatment according to the different profiles identified are detailed below. The group of hormone receptor-positive patients was divided into two subgroups, depending on the menopausal status,. However, we would like to point out that any patient with any tumor profile or at any disease stage may be considered for inclusion in a clinical trial, if this possibility exists.

### Patients with hormone receptor-positive and HER2-negative tumors

Approximately 67–70 % of all metastatic breast tumors contain estrogen and progesterone hormone receptor-positive cells [11, 12]. The initial endocrine treatment administered will vary according to the patient’s menopausal status.

Hormone therapy drugs administered in standard clinical practice include tamoxifen (selective estrogen receptor modulator) [13, 14], fulvestrant (selective estrogen receptor antagonist) [15, 16], and aromatase inhibitors (estrogen synthesis blockers). The latter, including letrozole and anastrozole [13], are non-steroidal and reversible, while other compounds, such as exemestane, are steroidal and bind irreversibly [17]. They are used alone or in association with luteinizing hormone-releasing hormone (LHRH) analogs [18–20] or anti-target therapies, such as everolimus [17, 21]. Everolimus is an mTOR complex inhibitor which modulates cell growth and proliferation and can reverse resistance to hormone therapy.

For this patient profile, the hormone treatment for pre-menopausal and post-menopausal subgroups will be defined first, and then, the chemotherapy indications will be described.

#### Hormone therapy in pre-menopausal patients

In this subgroup, the aim of hormone therapy is to achieve post-menopausal hormone levels in the pre-menopausal patient. If the patient is diagnosed with metastasis de novo, or she has had a DFS of 12 months or more and few symptoms, recommended the first-line treatment is ovarian ablation along with tamoxifen (SEOM level of certainty: high; strength of recommendation: A), or an aromatase inhibitor, which increases the clinical benefit with an acceptable safety profile (ESMO Categories of Evidence and Consensus: IA). A meta-analysis of the combination of tamoxifen and ovarian ablation showed a significant increase in overall survival (OS) (HR 0.78; *p* = 0.002) [18] and ovarian ablation combined with aromatase inhibitor showed a median time to progression (TTP) of 12 months [19].

In the second-line hormone treatment, or for patients with DFS of less than 12 months after treatment, ovarian ablation is recommended (SEOM level of certainty: low; strength of recommendation: B). This may be combined with an aromatase inhibitor [19], fulvestrant [20] or tamoxifen [18], depending on the patient’s previous treatment (ESMO Categories of Evidence and Consensus: IB) (SEOM level of Certainty: low; strength of recommendation: B). Fulvestrant and ovarian ablation has shown a median TTP of 6  months and a median OS of 32 months [20].

**Fig. 2:**
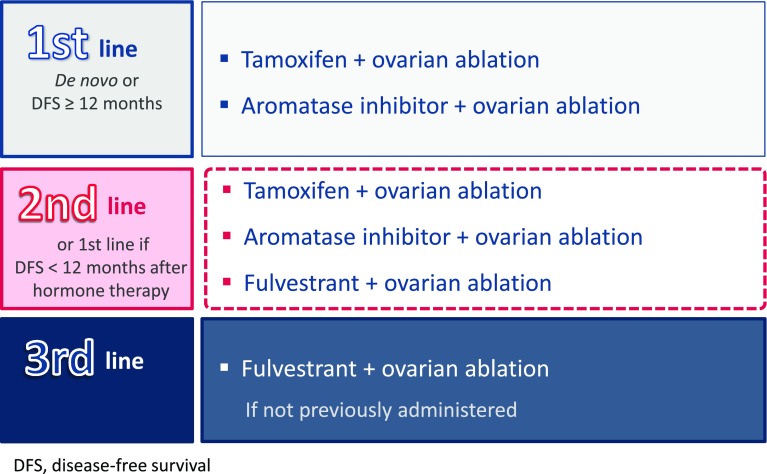
Proposed hormone therapy for pre-menopausal patients with hormone receptor-positive disease

Finally, for the third-line treatment, we recommend ovarian ablation and fulvestrant [20], if this drug has not been previously used (Fig. 2).

#### Hormone therapy in post-menopausal patients

In the first-line hormone therapy in post-menopausal patients (including cases of visceral metastasis, in the absence of visceral crisis), aromatase inhibitors are recommended, providing a median progression-free survival (PFS) of between 8 and 10 months, an objective response rate (ORR) of 33–46 %, and clinical benefit around 55 % [13, 15, 24] (ESMO Categories of Evidence and Consensus: IA) (SEOM level of certainty: high; strength of recommendation: A). In patients diagnosed with recurrence during adjuvant treatment with an aromatase inhibitor or who have a DFS of less than 12 months, treatment with fulvestrant is recommended [15, 24], (ESMO Categories of Evidence and Consensus: IB) (SEOM level of certainty: moderate; strength of recommendation: B), or the combination of exemestane and everolimus [17, 21], depending on the patient’s clinical status (ESMO Categories of Evidence and Consensus: IB). Efficacy data for fulvestrant show a TTP of 23.4 months and an OS of 54 months [15, 24], while for the combination of exemestane and everolimus, median PFS during the centralized evaluation was 10.6 months [17, 21]. In the sub-analysis by subgroups, median PFS for patients that had progressed to adjuvant treatment before 12 months was 15.2 months.

If the patient responded to the first-line treatment with an aromatase inhibitor, fulvestrant is recommended as the second line (median PFS 4.8 months [16]), or in the absence of symptoms of visceral disease, another option is the combination of exemestane and everolimus, which improves PFS [17, 21] (ESMO Categories of Evidence and Consensus: IA) (SEOM level of certainty: high; strength of recommendation: A).

**Fig. 3:**
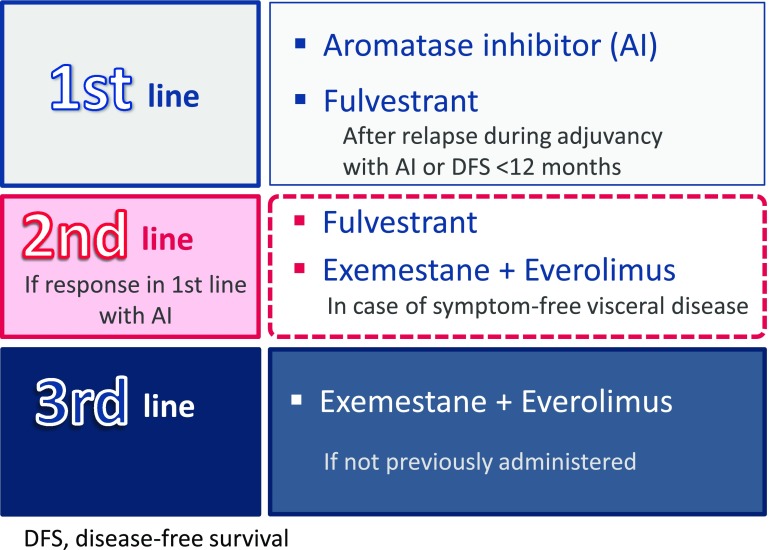
Proposed hormone therapy for hormone receptor-positive patients post-menopause or after ovarian ablation

In the third-line endocrine therapy, exemestane combined with everolimus [17] is recommended, if not used previously (Fig. 3).

#### Chemotherapy in patients with hormone receptor-positive disease

Chemotherapy for hormone receptor-positive patients includes microtubule inhibitors, such as taxanes (docetaxel [25], paclitaxel [26], and nab-paclitaxel [27], a nanoparticle albumin-bound paclitaxel), which are tubulin depolymerization inhibitors. Their mode of action consists of stabilizing GDP-bound tubulin in the microtubule. Other microtubule inhibitors are the vinca alkaloid vinorelbine [28], which is a tubulin polymerization inhibitor, and eribulin [29], a synthetic macrocyclic analog which binds to the positive growing end of the microtubules, suppressing dynamic instability.

Anthracyclines, such as doxorubicin and epirubicin, and pegylated liposomal doxorubicin [30], are cytotoxic agents that inhibit topoisomerase II, and DNA/RNA synthesis and generate oxygen free radicals. Similarly, gemcitabine is a pyrimidine antimetabolite that inhibits DNA synthesis [31], capecitabine acts as a thymidylate synthase inhibitor, and cyclophosphamide is an alkylating agent that interferes with DNA replication [32]. New agents include bevacizumab, a humanized anti-vascular endothelial growth factor (VEGF) monoclonal antibody [33].

In the case of aggressive disease, combined chemotherapy, such as paclitaxel plus bevacizumab, must be considered in the first-line chemotherapy treatment [33] when a rapid tumor response is desired (SEOM level of certainty: moderate; strength of recommendation: C). Treatment with anthracyclines and taxanes, either sequential or in combination [34, 35], may also be evaluated in this situation (SEOM level of certainty: high; strength of recommendation: A), including liposomal anthracyclines (median PFS 6.9 months and median OS 21 months in the first line [30]). Nab-paclitaxel is also an option for patients with taxane hypersensitivity. It has shown a median PFS of 13 months and a median OS of 33.8 months [36]. Another possibility in this situation is to use a combination of paclitaxel and gemcitabine [31], which has shown an improvement of TTP and OS (6.1–18.6 months, respectively).

For the first-line therapy in all other cases, monotherapy can be started with weekly paclitaxel (median PFS 5.9 months and median OS 25.2 months [26, 33]) or oral drugs, such as vinorelbine [28, 37] (median PFS 4.2–4.4 months and median OS of 16.4–24 months) or capecitabine [32, 38] (median TTP 4.1 months and median OS 19.6 months), according to patient preference (ESMO Categories of Evidence and Consensus: IA). Paclitaxel can also be used in combination with bevacizumab (median PFS 11.8 months and median OS 26.7 months [33]) or sequentially with anthracyclines (SEOM level of certainty: high; strength of recommendation: A) [34, 35]. In addition, for elderly patients or those in special situations, the use of metronomic regimens should be evaluated [41] (SEOM level of certainty: moderate; strength of recommendation: B).

In the second-line therapy, the use of capecitabine [32, 38], vinorelbine [28, 37], nab-paclitaxel (median OS 14 months [27]), or eribulin (median OS 15.2 months [29, 42]) is recommended in both pre- and post-menopausal patients, depending on the regimen administered in the first line (SEOM level of certainty: high; strength of recommendation: A). Other options include weekly paclitaxel [26] or liposomal anthracyclines [30]. Again, metronomic regimens (cyclophosphamide or vinorelbine) should be considered for elderly patients or those in special situations [41]. Vinorelbine has been studied in elderly patients, obtaining an ORR of 38 %, a median PFS of 7.7 months, and an OS of 15.9 months [43], and cyclophosphamide obtained an ORR of 31 % [44].

**Fig. 4:**
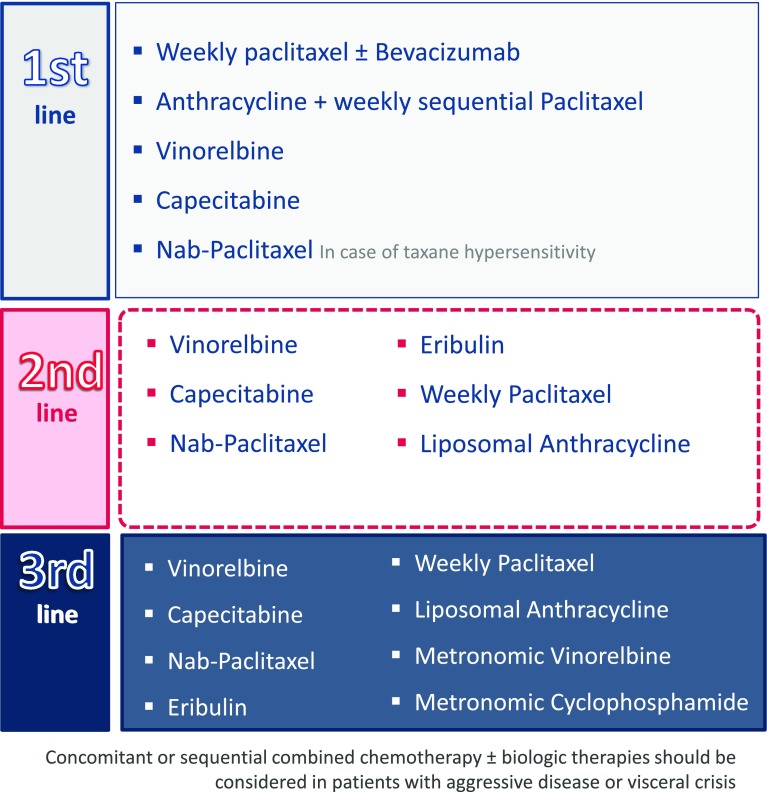
Proposed chemotherapy treatment for patients with hormone receptor-positive disease

Finally, third-line treatment in these cases consists of any of the above-listed options not previously used. At this time, metronomic regimens with vinorelbine [41, 43] or cyclophosphamide [41, 44] may be considered for all patients, not only the frail and the elderly, depending on their expectations and wishes (Fig. 4).

### Patients with HER2-positive disease

HER2 is an external membrane receptor of the breast cells that regulates proliferation. It has tyrosine kinase activity and is the product of the ERBB2 oncogene. HER2-positive disease accounts for 15–20 % of all breast cancers and is associated with a more aggressive natural history [11, 12].

A series of anti-HER2 drugs targeting this protein have been developed. These include the monoclonal antibodies, trastuzumab and pertuzumab, which bind to an extracellular component of HER2 [45]. Another compound is lapatinib, an intracellular tyrosine kinase inhibitor, which blocks the receptor signaling cascade [46]. Finally, the antibody–drug conjugate (adc) ado-trastuzumab emtansine (T-DM1) works in two ways: by disrupting HER2 signaling and by causing direct cytotoxicity [39]. Treatment of HER2-positive breast cancer generally consists of anti-HER2 inhibitors combined with the conventional chemotherapy (ESMO Categories of Evidence and Consensus: IA) (SEOM level of certainty: high; strength of recommendation: A).

**Fig. 5:**
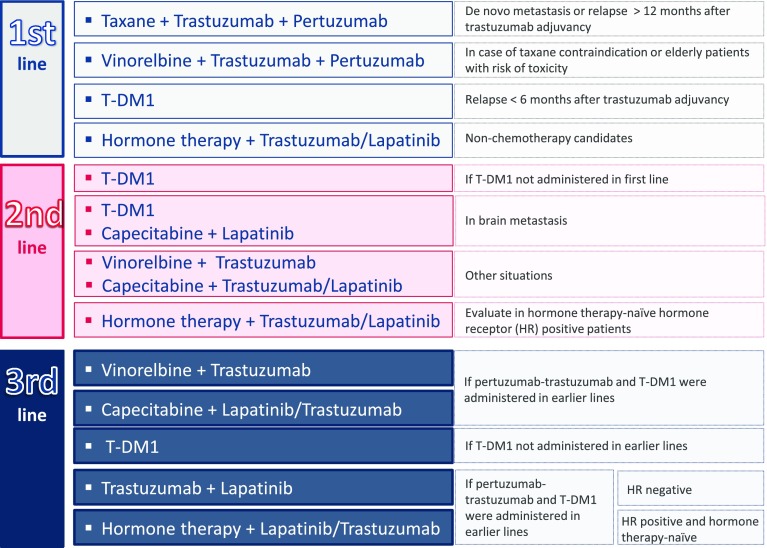
Proposed treatment for patients with HER2-positive breast cancer

As can be seen in Fig. 5, the first-line treatment in the case of de novo metastasis (SEOM level of certainty: high; strength of recommendation: A) or relapse after the first year following the end of adjuvant treatment with trastuzumab [47] (SEOM level of certainty: moderate; strength of recommendation: B) consists of a triple combination of taxanes with trastuzumab and pertuzumab. This combination has increased OS (56.5 months) and PFS (18.7 months) [45, 48] (ESMO Categories of Evidence and Consensus: IA). If taxanes are contraindicated, they should be replaced by vinorelbine in the same combination, which has shown a median PFS of 11.4–14.3 months [49, 50] (SEOM level of certainty: low; strength of recommendation: C). This same regimen should be considered for elderly patients with a risk of taxane toxicity. Generically, combining anthracyclines with trastuzumab is not recommended, since it increases the risk of cardiotoxicity [52] If the patient relapses within the first 6 months after completing the trastuzumab adjuvant treatment, the first-line treatment with T-DM1 is recommended, since it has shown a median PFS and OS of 15.2–29.8 months, respectively, and appears to be less toxic than lapatinib combined with capecitabine [39, 53] (SEOM level of certainty: moderate; strength of recommendation: B).

In hormone receptor-positive patients who are not candidates for chemotherapy, the administration of endocrine therapy combined with trastuzumab must be evaluated [54] (ESMO Categories of Evidence and Consensus: IA) (SEOM level of certainty: moderate; strength of recommendation: B). This recommendation is based on the results of the eLEcTRA study, which found that this treatment offered a median TTP of 14.1 months [55]. Hormone therapy combined with lapatinib also improves the median PFS (8.2 months) and clinical benefit rate (48 %) [56].

T-DM1 is recommended for the second-line treatment with a median PFS of 9.6 months and a median OS of 30.9 months [39] (ESMO Categories of Evidence and Consensus: IA) (SEOM level of certainty: high; strength of recommendation: A). Although the EMILIA study was not restricted to patients with brain metastases, this compound has shown activity in this subgroup of patients (median PFS 5.9 months and median OS 26.8 months [57]). Similarly, the combination of capecitabine and lapatinib improves survival outcomes in this subgroup [57, 58] (SEOM level of certainty: moderate; strength of recommendation: B). Other options in the second line could be to combine vinorelbine with trastuzumab (median TTP 15.3 months and median OS 38.8 months) [59, 60], or capecitabine with trastuzumab (median PFS 8.1 months and OS 27.3 months) [61] or capecitabine with lapatinib (median PFS 8.4 months and OS around 19 months) [62]. For the hormone therapy-naïve patients with hormone receptor-positive disease, hormone therapy combined with lapatinib [56] or trastuzumab [55] may be an option (SEOM level of certainty: moderate; strength of recommendation: B).

Third-line options depend on which regimens have already been administered. If the patient has received pertuzumab with trastuzumab and T-DM1, the recommendation is to combine trastuzumab with vinorelbine, a combination which causes fewer adverse effects than docetaxel [59, 60]. Another option is to combine capecitabine with lapatinib [46, 61] or trastuzumab (ESMO Categories of Evidence and Consensus: IB), which has shown good response rates in patients with progressive disease [61–63]. T-DM1 is recommended in patients who have not used it previously, with a median PFS of 6.2 months [64] (ESMO Categories of Evidence and Consensus: IB) (SEOM level of certainty: high; strength of recommendation: A). Finally, patients with hormone receptor-negative tumors may receive trastuzumab plus lapatinib. This combination improves clinical benefit, PFS and OS (14 months), compared to lapatinib alone [65] (SEOM level of certainty: moderate; strength of recommendation: B). If the cancer is hormone receptor-positive and the patient is hormone therapy-naïve, hormone treatment may be administered together with lapatinib [56] or trastuzumab [55] (SEOM level of certainty: moderate; strength of recommendation: B) (Fig. 5).

### Patients with triple-negative breast cancer

This group of patients constitutes approximately 10–20 % of breast cancer cases [66]. Triple-negative cancer is characterized by cells that express neither hormone nor HER2 receptors. Accordingly, treatment is based on the use of chemotherapy and biological therapies. Platinum compounds, widely used in the first line, include cisplatin and carboplatin, which are alkylating-like agents that interfere with DNA replication [67].

#### De novo metastasis or relapse after >12 months disease-free survival

**Fig. 6:**
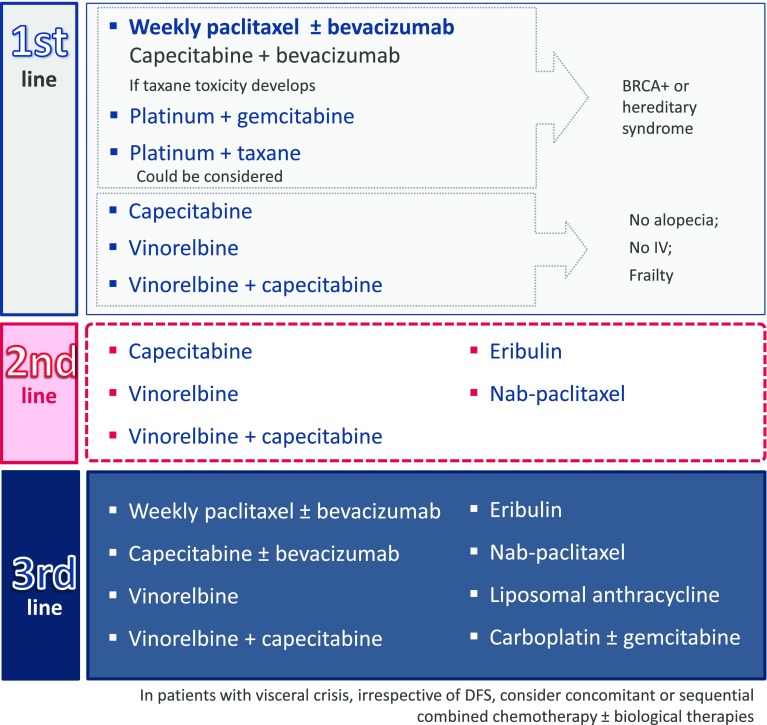
Proposed treatment for patients with triple-negative metastatic cancer de novo or with a progression-free survival greater than 12 months

In patients with de novo metastasis or DFS >12 months, the recommendation for the first-line treatment is weekly paclitaxel, either as a single agent (ESMO Categories of Evidence and Consensus: IA) or combined with bevacizumab (SEOM level of certainty: moderate; strength of recommendation: C). The combination has been reported to increase median PFS [33]. If the patient responds to treatment, but develops early toxicity, paclitaxel can be switched to capecitabine, but not as a maintenance treatment. This recommendation is based on a phase III study which found that the PFS and safety profiles of the two combinations should, in principle, be equivalent (median PFS 6.1 months for the capecitabine arm and 6.5 for the taxane arm) [68]. Another good option for frail patients or for those who cannot or do not want to receive intravenous chemotherapy is the oral administration of capecitabine [32, 38] and vinorelbine [28, 37] separately or in combination (median OS 22.2 months with a Disease Control Rate [DCR] of 70.5 %) [69]. This is also the preferred choice for patients who want to avoid alopecia (Fig. 6) (ESMO Categories of Evidence and Consensus: IA) (SEOM level of certainty: moderate; strength of recommendation: B).

BRCA-positive patients or hereditary syndrome may receive a platinum salt in monotherapy (ESMO Categories of Evidence and Consensus: IC) (SEOM level of certainty: moderate; strength of recommendation: B). A phase III study comparing carboplatin with docetaxel, both in monotherapy, achieved a median OS of 12.3–12.4 months, and a median PFS of 4.5–3.1 months, respectively, although in the mutated BRCA subgroup PFS was 6.8 months for the carboplatin arm and 4.8 months for the docetaxel arm [70]. The platinum salt can also be administered in combination with gemcitabine (SEOM level of certainty: low; strength of recommendation: B). In a global population, this combination has shown a 32 % ORR, a median PFS of 4.1 months, and a median OS of 11.1 months [71] The combination of a platinum salt with a taxane may be evaluated: high response rates have been reported (62 %) with a median TTP of 4.8 months and a median OS of 16 months [72]. Another alternative is a taxane combined with bevacizumab [33].

In the second line, the recommendation is to use capecitabine [32, 38], or vinorelbine [28, 37], or a combination of both. In this same setting, eribulin treatment has been shown to improve OS compared to other treatments selected according to the investigator’s criteria in a phase III trial and in the pooled analysis of the two available phase III studies [29, 42]. Another option would be to use nab-paclitaxel, which has shown greater efficacy and a better safety profile than tri-weekly paclitaxel [27] and tri-weekly docetaxel [36].

In the third-line treatment, any of the drugs of the previously mentioned lines that have not yet been used are recommended. Additional recommended options include liposomal anthracycline [30] and carboplatin, with or without gemcitabine [70, 71]. The results of a phase III trial suggest that liposomal anthracycline is equally effective as standard anthracycline, but associated with lower cardiotoxicity [30]. Treatment regimens are summarized in Fig. 6.

In patients with visceral crisis, irrespective of DFS, concomitant or sequential combined chemotherapy may be considered, with the possible addition of biological therapies.

#### Metastasis due to disease recurrence during the first 12 months

**Fig. 7:**
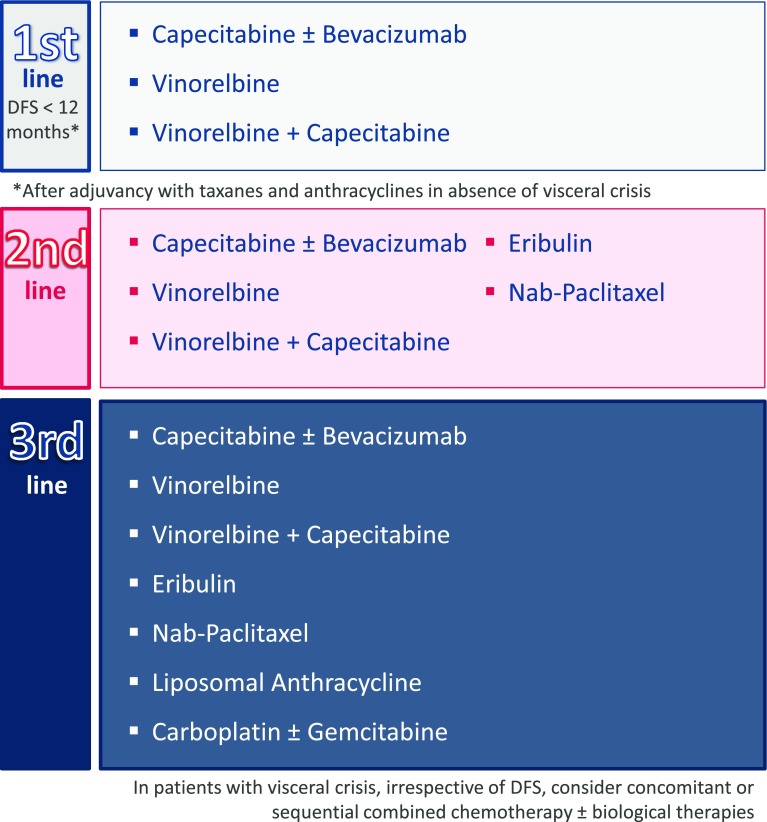
Proposed treatment for patients with triple-negative metastatic cancer with a progression-free survival less than 12 months

If a patient’s DFS lasts less than 12 months after adjuvant treatment with taxanes and anthracyclines, the options for the first-line treatment include vinorelbine (a good salvage therapy after failure on anthracyclines and taxanes, since it does not cross-react with taxanes [28, 37, 73]), and capecitabine (that moreover can be combined with bevacizumab [32, 38, 68] or vinorelbine [69]) (Fig. 7) (ESMO Categories of Evidence and Consensus: IB).

For the second-line therapy, eribulin [29, 42] or nab-paclitaxel [27, 36], are recommended, as well as capecitabine, in combination with bevacizumab; another option is vinorelbine separately or in combination with capecitabine, if not previously used. In the third line, we recommend any of the drugs of the previously mentioned lines that has not yet been used (Fig. 7), including liposomal anthracycline [30] or carboplatin as a single agent or in combination with gemcitabine [70, 71].

In patients with visceral crisis, irrespective of DFS, concomitant or sequential combined chemotherapy may be considered, with the possible addition of biological therapies.

## Future perspectives

The outlook for these patient profiles is evolving rapidly, and some new drugs are currently in development. Palbociclib, an oral small-molecule selective inhibitor of cyclin-dependent kinase 4/6, has been developed for patients with HER2-negative and hormone receptor-positive tumors. It was tested in PALOMA-1, a phase II trial comparing letrozole alone or in combination with palbociclib in previously untreated post-menopausal patients. Primary endpoint was PFS. The experimental arm obtained a median PFS of 20.2 vs 10.2 months in the placebo arm (HR 0.488; *p* = 0.004) [75]. PALOMA-3, the phase III trial, included pre- and post-menopausal patients who progressed on one prior endocrine therapy, with an aromatase inhibitor in the case of post-menopausal patients. These patients were randomized to receive fulvestrant combined with palbociclib or placebo. Efficacy results were recently reported. The study met its primary endpoint with a median PFS of 9.3 months for the experimental arm vs 4.6 months in the placebo arm (HR 0.46; *p* < 0.001) [76]. The FDA approved the indication of palbociclib in combination with fulvestrant after progression on a previous endocrine therapy in February, 2016 [77]. Currently, 666 patients are enrolled in PALOMA-2, a phase III trial comparing letrozole combined with palbociclib vs placebo in post-menopausal patients who are candidates for the first-line treatment with endocrine therapy. Study results have not yet been reported (NCT01740427).

Neratinib, an oral irreversible pan-ErbB receptor tyrosine kinase inhibitor, has been developed for patients with HER2-positive tumors. NEfERT-T is a randomized controlled phase II study which tested the combination of paclitaxel with neratinib or trastuzumab in the first line. Median PFS, the primary endpoint, was 12.9 months for both combinations (HR 1.02; *p* = 0.89). However, combination with neratinib reduces symptomatic CNS recurrences (HR 0.48; *p* = 0.002) and 2-year incidence of CNS metastasis (HR 0.45; *p* = 0.004) [78]. Another randomized phase II trial was designed to demonstrate the non-inferiority of single-agent neratinib in PFS vs capecitabine combined with lapatinib. Median PFS of neratinib was 4.5 vs 6.8 months for the combination arm. Median OS was also superior in the combination arm (19.7 vs 23.6 months) [79]. A phase I/II trial which tested the combination of capecitabine with neratinib showed a promising ORR of 64 % in patients not previously exposed to lapatinib, and 57 % in previously exposed patients. Median PFS was 40.3–35.9 weeks, respectively [80]. Currently, a phase III trial comparing capecitabine plus neratinib or lapatinib is recruiting patients who have previously received with at least two anti-HER2 therapies. This study is exploring two co-primary endpoints, PFS and OS (NCT01808573).

In patients with triple-negative tumors, olaparib, a novel orally active poly(ADP-ribose) polymerase (PARP) inhibitor which induces synthetic lethality in BRCA-deficient cells is being developed in breast cancer with BRCA 1/2 mutations. It is currently indicated in patients with platinum-sensitive relapse of a BRCA-mutated ovarian cancer. In BRCA-mutated breast cancer, a phase II trial was developed with the inclusion of 54 patients. The first cohort of 27 patients received olaparib 400 mg twice daily and the second cohort of 27 patients received 100 mg twice daily. ORR, the primary endpoint, was 41 % in the first cohort and 22 % in the second one [81]. A phase III trial, OlympiAD, is now being developed. Patients with pre-treated BRCA 1/2 mutated breast cancer were randomized to receive olaparib in monotherapy or their physicians’ choice of chemotherapy. Results of this study have not yet been reported (NCT02000622).

## Conclusion

Three MBC patient profiles have been outlined in this document. They have been classified according to genomic characteristics, and subgroups have been formed according to DFS and pre- and post-menopausal status, which can be treated differently.

In their clinical practice, the specialists we consulted reserve chemotherapy for the treatment of patients with triple-negative breast cancer, aggressive hormone receptor-positive disease, visceral crisis, or hormone therapy resistance. It is also used to complement biological therapy in patients with HER2-positive disease.

Patients with hormone receptor-positive tumors who do not have aggressive disease or visceral crisis receive endocrine therapy as a first option and up to three lines of treatment may be administered.

In patients who are already hormone-resistant or who have more aggressive disease than candidates for endocrine therapy, but not as aggressive as candidates for combined chemotherapy, the treatment of choice may be single-agent therapy with vinorelbine or capecitabine, but anthracycline or paclitaxel must also be taken into consideration. In addition, in the case of aggressive disease or visceral crisis, paclitaxel can be used in combination with bevacizumab.

In patients with HER2-positive disease, the recommendation is to combine a taxane with dual anti-HER2 blockade with trastuzumab and pertuzumab. If taxanes are contraindicated, vinorelbine can be used instead. All patients with HER2-positive disease can be treated with T-DM1 in any of the lines (in the case of the first-line treatment, those with relapse diagnosed during the first 6 months after completing adjuvancy).

For patients with triple-negative cancer, a common first-line option is weekly paclitaxel, with or without bevacizumab, unless resistance has developed (DFS < 12 months), in which case capecitabine or vinorelbine may be administered. In the second line, eribulin or nab-paclitaxel are generally given, and in the third line, liposomal anthracycline and carboplatin combined with gemcitabine are added to the list of choices.
